# Audit of Current Practise of Transfer of Care From Child and Adolescent Mental Health Services (CAMHS) to Adult Mental Health Services (AMHS) in Devon Partnership NHS Trust

**DOI:** 10.1192/bjo.2025.10565

**Published:** 2025-06-20

**Authors:** Lucia Curran

**Affiliations:** Devon Partnership NHS Trust, Devon, United Kingdom. Child and Family Health Devon, Devon, United Kingdom

## Abstract

**Aims:** The transfer of care from CAMHS to AMHS is often poorly managed which is distressing for young people and their families. The implications of poor transition include disengagement from services and deterioration in young people’s mental health.

In Devon Partnership NHS Trust (DPT) the transfer of care standard operating procedure (SOP) outlines 8 core standards of transition including clarification of clinical responsibilities, proposed timelines for task completion and documentation requirements. This audit compared DPT patient data against these core standards.

We aimed for 100% compliance between current practice in the transfer of care of patients from CAMHS to AMHS in North Devon and the recommended practice laid out in DPT’s SOP.

**Methods:** Data was collected via retrospective review of electronic patient notes of 51 young people aged 18–25 years old that presented to North Devon Liaison or Home Treatment Teams between 01/05/2024–01/08/2024.

28 participants (55% of the original cohort) were formerly known to CAMHS. 12 participants (43% of the former CAMHS sub-cohort) underwent transfer of care to AMHS. Data was collected on these 12 participants comparing case notes to SOP transition standards.

**Results:** There were evident strengths of current transition practices demonstrated by 100% of CAMHS specialist service users at the time of transition securing AMHS input and 57% of those referred for transition were issued a care plan with a defined exit from CAMHS.

Weaker areas included only 14% of young people receiving explanation as to why services could not be offered and only 14% were allocated a doctor with medical responsibility on transfer. There was a disappointing lack of collaboration between services as only 29% had a documented joint meeting between CAMHS and AMHS.

**Conclusion:** There is certainly room for improvement in current transfer of care practices in DPT. Hopefully this audit generates discussions and reconsideration of current practices to initiate change at which point a re-audit could be conducted. Ultimately it is hoped to improve the level of care for young people at a vulnerable time of change in their care provision between CAMHS and AMHS.

